# Impact of Antibiotic and Steroid Therapy on Leptospirosis Outcomes: A Retrospective Cohort Study in Transcarpathia, Ukraine

**DOI:** 10.3390/biomedicines12081685

**Published:** 2024-07-29

**Authors:** Pavlo Petakh, Mykhailo Poliak, Anton Kohutych, Valentyn Oksenych, Oleksandr Kamyshnyi

**Affiliations:** 1 Department of Biochemistry and Pharmacology, Uzhhorod National University, 88000 Uzhhorod, Ukraine; pavlo.petakh@uzhnu.edu.ua; 2 Department of Microbiology, Virology and Immunology, I. Horbachevsky Ternopil National Medical University, 46001 Ternopil, Ukraine; 3 Transcarpathian Regional Clinical Infectious Hospital, 88000 Uzhhorod, Ukraine; mykhailo.polyak@uzhnu.edu.ua (M.P.); a.kohutych@uzhnu.edu.ua (A.K.); 4 Broegelmann Research Laboratory, Department of Clinical Science, University of Bergen, 5020 Bergen, Norway

**Keywords:** leptospirosis, corticosteroids, outcome, antibiotic, dexamethasone, predictors

## Abstract

Leptospirosis presents a significant health challenge in the Transcarpathian region of Ukraine, with higher incidence rates and mortality compared to national averages. We conducted a retrospective cohort study to investigate the effects of antibiotic and steroid treatments on outcomes in leptospirosis patients. Our analysis of clinical and laboratory data from a single center revealed that dexamethasone showed significant effects on various clinical variables, as did investigated antibiotics. Notable differences in clinical and laboratory outcomes were observed, particularly in direct bilirubin levels, which were significantly higher in non-survivors. ROC analysis demonstrated high sensitivity and specificity of direct bilirubin as a predictor of mortality. These findings highlight the importance of targeted treatment strategies and the potential of specific laboratory markers in improving leptospirosis management.

## 1. Introduction

Leptospirosis, a disease primarily found in tropical regions, is a zoonotic illness transmitted through contact with the urine of animals, particularly rats [1]. Its clinical manifestations range from mild to severe, posing life-threatening consequences. Symptoms often mimic those of other infectious diseases, such as influenza [2]. Severe cases, affecting approximately 5% to 15% of individuals, manifest with acute renal failure, acute respiratory distress syndrome, pulmonary issues, hypotension, icterus, and altered mental status [3,4,5,6,7].

In the Transcarpathian region, leptospirosis persists as a prevalent zoonotic disease. Between 2005 and 2015, 420 cases were reported, with an incidence three times higher than the national average. The case fatality rate (CFR) for leptospirosis in Transcarpathia averages 12.5%, exceeding the national level of 9.8% [8,9,10].

**Figure 1:**
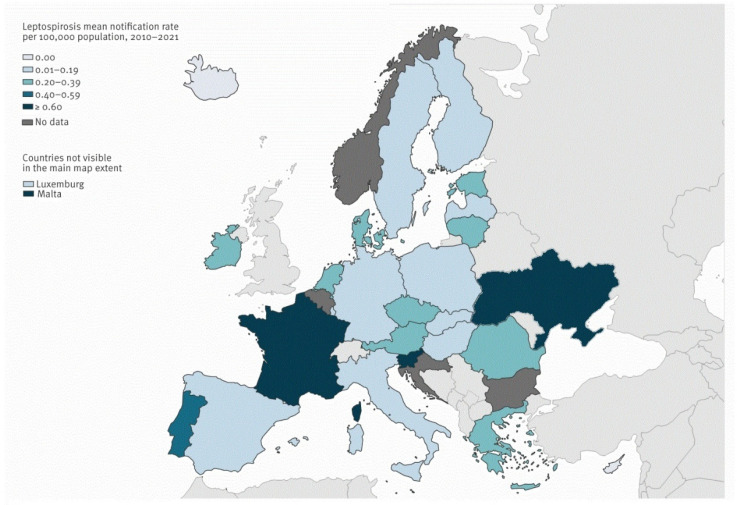
Average annual rate of confirmed leptospirosis cases per 100,000 population, European Union/European Economic Area, 2010–2021. Adapted from ECDC [12]. The average number of leptospirosis cases in Ukraine is one of the highest in Europe, amounting to 0.73 per 100,000 people for the period 2010–2021. In comparison, in neighboring countries, the rate is 0.1 in Poland, 0.15 in Slovakia, and 0.34 in Romania.

Analysis reveals a significant increase in the notification rate of leptospirosis in Ukraine in 2023. This surge is primarily attributed to the rising incidence of leptospirosis in Transcarpathia, accounting for 150 cases out of the total 433 in Ukraine, and the Ivano-Frankivsk region, contributing 34 cases [11] (Figure 1).

Pathogenic Leptospira, responsible for the disease, are excreted in the urine of rats, with humans inadvertently becoming hosts and facing potentially life-threatening consequences [7]. Rats, however, remain immune to fatal infection, serving as natural reservoirs. Most human leptospiral infections are either mild or asymptomatic. Those who do develop the illness typically experience an abrupt onset of symptoms, including fever, rigors, myalgias, and headache [6,9].

The disease unfolds in two phases [13]. The initial phase involves an acute febrile bacteremia lasting 2 to 9 days, followed by a period of reduced or no fever and apparent improvement. The second phase, known as the “immune” phase, is characterized by renewed fever and the emergence of complications. Approximately 5–15% of patients may progress to Weil’s disease, with pulmonary involvement being a notable feature (20–70%) [7]. Pulmonary complications range from mild cough to severe symptoms like hemoptysis and acute respiratory distress syndrome (ARDS), the latter carrying a high mortality rate of around 50% [14,15,16].

The role of corticosteroids in treating severe leptospirosis, especially in addressing pulmonary complications like ARDS, has been explored in a limited number of studies. The argument posits that multi-organ failure in leptospirosis may result from an overactive immune system rather than the direct effects of the pathogen [17]. Therefore, the use of therapeutic doses of steroids is considered to counteract immune activation, potentially reducing mortality and morbidity in severe leptospirosis cases [18]. However, the efficacy of corticosteroids remains uncertain due to the scarcity of studies.

This study aims to investigate the effects of antibiotic and steroid treatment on outcomes in patients diagnosed with leptospirosis within the Transcarpathian Region of Ukraine. Through a retrospective analysis of clinical and laboratory data from a single-center cohort, we aim to assess the impact of these treatments on patient morbidity and mortality rates. Additionally, we aim to explore potential associations between treatment regimens and clinical outcomes, thereby providing significant contributions to the understanding and management of leptospirosis in this geographical area.

## 2. Materials and Methods

### 2.1. Study Design and Diagnostic Criteria

We conducted a retrospective single-center cohort study. All medical records were obtained from the Transcarpathian Regional Clinical Infectious Diseases Hospital, Ukraine. Confirmation of the diagnosis was determined by the order of the Ministry of Health of Ukraine No. 905 of 28 December 2015. This includes fever or at least two of the following symptoms: chills, headache, myalgia, conjunctival hyperemia, skin and mucous membrane hemorrhages, rash, jaundice, myocarditis, meningitis, kidney failure, respiratory manifestations such as hemoptysis, and laboratory confirmation by Polymerase Chain Reaction (PCR) or MAT (Microscopic Agglutination Test).

Inclusion Criteria: Patients included in this study met the following criteria: diagnosed with leptospirosis according to the criteria outlined by the Ministry of Health of Ukraine; medical records available at the Transcarpathian Regional Clinical Infectious Diseases Hospital; confirmed laboratory diagnosis by PCR or MAT; complete clinical and laboratory data for analysis; aged 18 years and older; and non-pregnant.

Exclusion Criteria: Patients were excluded from the study if they met any of the following criteria: incomplete medical records or missing key diagnostic information; incomplete or inconsistent laboratory confirmation results; history of receiving medications that could interfere with the study variables or outcomes; younger than 18 years old; or pregnant.

### 2.2. Patient Stratification and Grouping

Randomized Controlled Trials (RCTs) and observational cohort studies We have grouped these patients into different groups for comparing clinical and laboratory data. Firstly, we grouped them based on sex into males (n = 27) and females (n = 11). Secondly, based on steroid treatments, the majority received dexamethasone at 8 mg per day (n = 29), while a few received methylprednisolone at 250 mg per day (n = 2), and others included patients prescribed prednisolone at 1 mg/kg/day or combination steroid types (n = 4), with 3 patients not receiving steroids. Next, we grouped the patients based on antibiotic treatment into large groups, i.e., those treated with cephalosporins (n = 17), benzylpenicillins (n = 8), and others treated with carbapenems or macrolides (n = 13). Cephalosporins and benzylpenicillin were the primary treatments, while carbapenems and macrolides were used as alternatives. This categorization facilitates a detailed comparison of their clinical efficacy and laboratory impact. Patients were prescribed infusion therapy, and, if appropriate, specific treatment for their comorbid conditions. Finally, the patients were also grouped based on outcomes, i.e., 35 survivors and 3 non-survivors.

### 2.3. Statistical Analysis

Power analysis was conducted to ascertain the appropriate sample size necessary to detect statistically significant effects or differences in our study variables while maintaining a desired level of statistical power. The power analysis confirmed that a sample size of 38 patients was adequate to detect significant differences with 80% power and an alpha level of 0.05. Effect sizes for key outcomes were calculated, with notable findings such as a Cohen’s d of 1.2 for the difference in direct bilirubin levels between survivors and non-survivors.

Quantitative variables following a non-normal distribution were described using the median (Me) and lower and upper quartiles (Q1–Q3). Categorical data were described using absolute and relative frequencies. Comparisons of three or more groups on a quantitative variable with a distribution differing from normality were made using the Kruskal–Wallis test, with Dunn’s criterion and Holm correction applied as a post hoc method. The Wilcoxon test was employed to compare quantitative variables following a non-normal distribution between two matched samples. The Mann–Whitney U-test was used to compare two groups on a quantitative variable with a distribution differing from normality. Frequencies in the analysis of multifield contingency tables were compared using Pearson’s chi-square test.

ROC analysis was utilized to assess the diagnostic performance of quantitative variables in predicting a categorical outcome. The optimal cut-off value of the quantitative variable was estimated using Youden’s J statistic. Statistical significance was determined at *p* < 0.05.

## 3. Results

### 3.1. Descriptive Analysis and Sex-Dependent Changes in Variables

Overall, 38 patients were included in our study, of whom 35 survived and 3 died (7.89%). Survivors were generally younger (median age 48) and had fewer co-morbid conditions compared to non-survivors (median age 65). The socio-demographic and clinical presentations and laboratory findings of all confirmed patients are shown in Table 1.

The 38 patients comprised 27 (71.1%) males and 11 (28.9%) females, with a median age of 50.00 years (Table 2). Additionally, we conducted a sex-dependent investigation of clinical and laboratory variables on admission. We found significant changes in Mean Corpuscular Volume (MCV) (*p* = 0.002), with males having 91.00 (88.50–94.30) fL and females having 87.00 (83.50–88.50) fL. Males had statistically higher Alanine Aminotransferase (ALT) levels (*p* = 0.0048) than females, with values of 93.30 (65.20–134.15) U/L compared to 61.50 (38.75–100.55) U/L, respectively. Similarly, males had higher Aspartate Aminotransferase (AST) levels (*p* = 0.020) at 81.30 (54.90–148.95) U/L compared to females at 47.00 (30.55–73.55) U/L.

### 3.2. Effect of Steroid Type on Clinical and Laboratory Findings

Most patients took dexamethasone, comprising 29 out of 38 patients (76.3%). Additionally, only two patients (5.3%) received methylprednisolone. Four patients were administered other types of steroids, while three patients did not receive any steroid treatment during their hospitalization.

**Figure 2:**
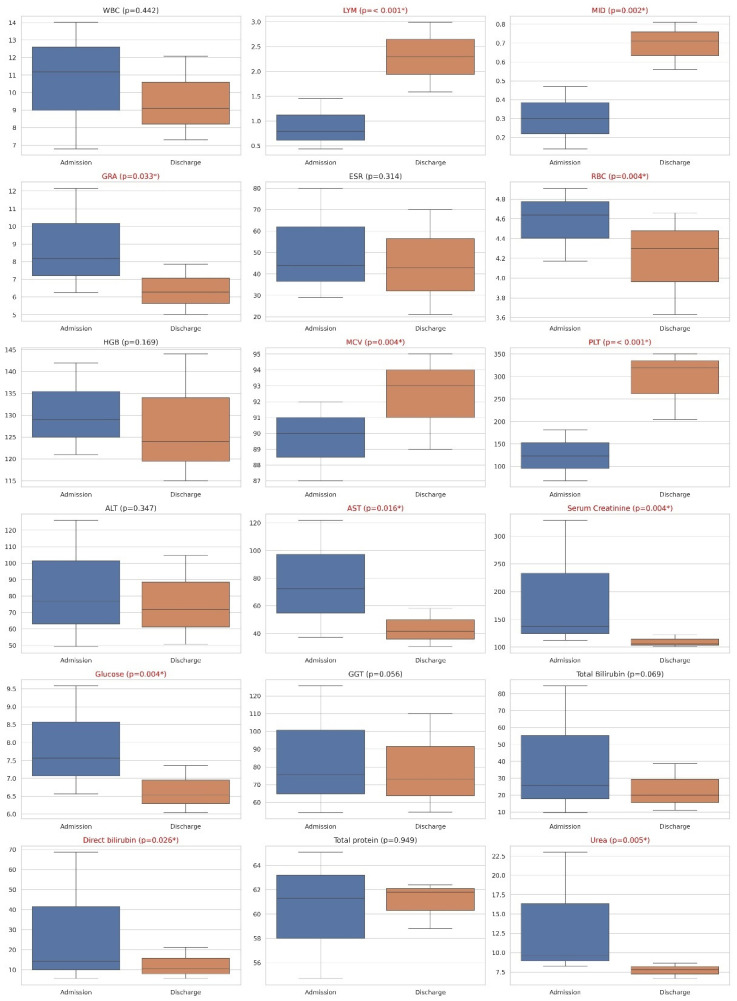
Facet box plots of laboratory findings on admission and discharge in patients who took dexamethasone. Statistically significant changes were observed in multiple parameters including LYM, MID, GRA, RBC, MCV, PLT, AST, direct bilirubin, and urea levels. Variables marked in red and with an asterisk (*) indicate statistically significant changes between admission and discharge.

When we compared laboratory findings before admission and after discharge, we found statistically significant changes only in the group that took dexamethasone. The statistically significant changes were observed in lymphocytes (LYM) levels (on admission: 0.79 × 10^9^/L (0.44–1.45), on discharge: 2.30 × 10^9^/L (1.59–2.99), *p* < 0.001), mid-sized cells (MIDs) (on admission: 0.30 × 10^9^/L (0.14–0.47), on discharge: 0.71 × 10^9^/L (0.56–0.81), *p* = 0.002), granulocyte (GRA) levels (on admission: 8.18 ×10^9^/L (6.25–12.16), on discharge: 6.28 × 10^9^/L (5.00–7.87), *p* = 0.033), red blood cells (RBCs) (on admission: 4.64 × 10^12^/L (4.17–4.91), on discharge: 4.30 × 10^12^/L (3.63–4.66), *p* = 0.004), MCV (on admission: 90.00 fL (87.00–92.00), on discharge: 93.00 fL (89.00–95.00), *p* = 0.004), platelet (PLT) levels (on admission: 123.00 × 10^9^/L (68.00–182.00), on discharge: 320.00 × 10^9^/L (204.00–350.00), *p* < 0.001), AST (on admission: 72.30 U/L (37.30–122.00), on discharge: 41.50 U/L (30.50–58.10), *p* = 0.016), direct bilirubin (on admission: 14.40 µmol/L (5.68–68.70), on discharge: 10.50 µmol/L (5.70–21.20), *p* = 0.026), and urea levels (on admission: 9.63 mmol/L (8.22–23.02), on discharge: 7.80 mmol/L (6.65–8.63), *p* = 0.005). All other laboratory parameters such as white blood cells (WBCs) (×10^9^/L), the erythrocyte sedimentation rate (ESR) (mm/h), hemoglobin (HGB) (g/L), ALT (U/L), serum creatinine (µmol/L), glucose (mmol/L), Gamma-Glutamyl Transferase (GGT) (U/L), total bilirubin (µmol/L), and total protein (g/L) did not show significant differences between admission and discharge (*p* > 0.05) (Figure 2).

### 3.3. Effect of Antibiotic Treatment on Clinical and Laboratory Findings

We divided patients into three groups, i.e., those who were prescribed cephalosporins with 17 patients, those who were prescribed benzylpenicillin with 8 patients, and a group receiving alternative antimicrobial therapy with 13 patients. We compared the differences in the same indicators as when comparing the effects of steroids.

**Figure 3:**
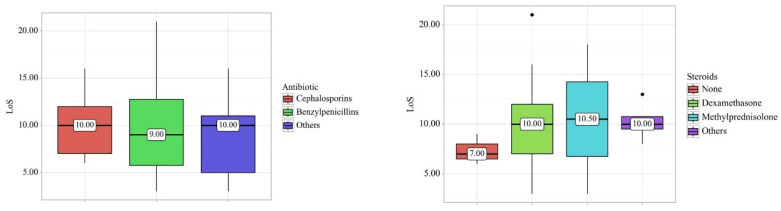
Box plots illustrating the effect of steroids and antibiotics on Length of Stay (LoS). No significant changes were observed in patients receiving either steroids or antibiotics.

When comparing laboratory findings before admission and after discharge, we observed statistically significant changes in LYM (*p* = 0.002), MID (*p* = 0.027), MCV (*p* = 0.006), PLT (*p* = 0.003), AST (*p* = 0.015), glucose (*p* = 0.045), total bilirubin (*p* = 0.009), direct bilirubin (*p* = 0.015), and urea (*p* = 0.013) for the group treated with cephalosporins. Patients treated with benzylpenicillins exhibited statistically significant changes in LYM (*p* = 0.039), MID (*p* = 0.028), RBC (*p* = 0.039), HGB (*p* = 0.028), and PLT (*p* = 0.039). Patients receiving alternative antimicrobial therapy experienced significant changes in LYM (*p* = 0.033), RBC (*p* = 0.008), MCV (*p* = 0.033), PLT (*p* < 0.001), Serum Creatinine (*p* < 0.001), direct bilirubin (*p* = 0.048), and urea (*p* = 0.010) (Table 3). When we compared the effect of antibiotics and steroids on the length of stay (LoS) and outcomes, we did not find any significant differences across the groups (Figure 3).

### 3.4. Effect of Laboratory Variables on Outcome

The non-survivor group had statistically higher serum creatinine levels on admission compared to the survivor group (137.30 [111.80–321.90] vs. 541.30 [506.10–570.60], *p* = 0.032) and GGT levels (75.60 [49.85–125.55] vs. 185.30 [163.75–442.35], *p* = 0.032). Total bilirubin levels were significantly higher in the non-survivor group compared to the survivor group (491.60 [343.95–563.30] vs. 25.90 [11.35–82.76], *p* = 0.010), as were direct bilirubin levels (257.10 [199.30–398.85] vs. 14.40 [5.84–53.80], *p* = 0.010). Additionally, urea levels were higher in the non-survivor group (30.80 [30.59–38.15] vs. 9.63 [8.20–21.25], *p* = 0.019).

**Figure 4:**
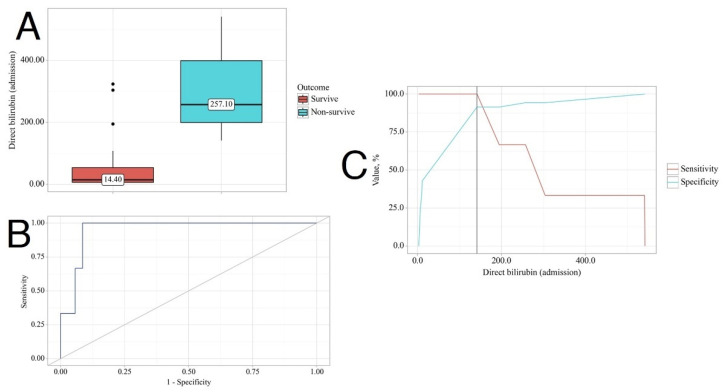
Direct bilirubin levels on admission as a potential predictor of lethality in leptospirosis. (**A**). Box plot illustrating direct bilirubin levels on admission in surviving and non-surviving patients. (**B**). ROC curve depicting the relationship between the probability of outcome and direct bilirubin. (**C**). Analysis of sensitivity and specificity of outcome based on direct bilirubin levels.

Moreover, we conducted a Receiver Operating Characteristic (ROC) analysis. We found that all variables had 100% sensitivity as potential predictors of death, except for GGT, which had a sensitivity of 66.8%. The specificity was lowest for GGT (77.1%), followed by serum creatinine (85.7%), total bilirubin, and urea (both 88.6%), and the highest specificity was for direct bilirubin (91.4%) (Figure 4).

## 4. Discussion

Although leptospirosis is less prevalent in Ukraine than in regions with subtropical climates, it is still reported annually. According to the Public Health Centre of Ukraine, there were 295 registered cases of leptospirosis in 2019 (0.7 per 100,000), 120 cases in 2020 (0.28 per 100,000), 122 cases in 2021 (0.29 per 100,000), 141 cases in 2022 (0.34 per 100,000), and 433 cases in 2023 (1.06 per 100,000) [11]. However, the actual incidence is likely higher due to underdiagnosis and under-reporting, as noted by Zubach et al. [19].

The Transcarpathian region experienced a significantly higher notification rate in 2023, with 150 out of 433 reported cases originating from this area. Situated in the western part of Ukraine, the region shares borders with Romania, Hungary, Poland, and Slovakia [8]. It sustains an above-average human population density, with 63% residing in rural regions, which may impact notification rates [20].

Leptospiral cases increased in 2023 to 292 cases compared to the previous year, 2022. The ongoing conflict in Ukraine poses a unique occupational risk to military personnel, who have an increased risk of infection due to exposure to contaminated water sources and potential reservoir hosts such as rodents [21]. The civilian population is also at risk following the destruction of the Kakhovka Dam in 2023, which has led to potential outbreaks of rodent-borne diseases, including leptospirosis and tularemia [22]. Zubach et al. reported a case of leptospirosis in a 70-year-old man in Lviv who was infected in bomb shelters [23]. This evidence suggests that leptospiral incidence will likely increase in the future.

We also compared the distribution of leptospiral serogroups in our regional datasets with available data from the Lviv Oblast. Our findings showed that the serogroup Hebdomadis was the most prevalent in our data at 26.3%, whereas the serogroup Icterohaemorrhagiae dominated in Lviv Oblast at 33.33%. Notably, the serogroup Grippotyphosa, the second most common in Lviv Oblast (25%), was absent in our data.

The serogroup Canicola showed similar prevalence in both datasets (our data: 10.5%; Lviv Oblast: 7.25%), and the serogroup Pomona was also comparable (7.9% vs. 8.33%). The serogroups Cynopteri and Sejroe were more prevalent in our region (18.4% and 13.2%, respectively) than in Lviv Oblast (3.62% and 1.09%, respectively) [24]. During the period from 2005 to 2015, the predominant serogroups in our Transcarpathian region were Icterohaemorrhagiae, Hebdomadis, and Grippotyphosa [8].

Recent results indicate that the Icterohaemorrhagiae serogroup is the most prevalent in Europe, accounting for 53% of cases, followed by the Australis serogroup at 13%. Additionally, the Pomona serogroup was identified in 5% of cases, the Autumnalis serogroup in 4%, and the Sejroe serogroup in 2% [25].

We observed that males had higher levels of transaminases such as AST, ALT, and GGT, but this did not affect outcomes. Our findings align with a study conducted in Germany, which revealed that male patients were more likely than female patients to be hospitalized and exhibit symptoms of severe leptospirosis, including jaundice, renal impairment, and hemorrhage [26].

Only four studies were found that investigated the role of steroids in clinical outcomes in leptospirosis. In three of these studies, methylprednisolone was administered at the initiation of treatment, albeit at varying doses. They suggested a potential beneficial role of steroids, particularly in patients with lung involvement [27,28,29]. However, the study that employed dexamethasone at the initiation failed to show a treatment benefit and, notably, reported an elevated incidence of nosocomial infections [30].

In regard to the effect of antibiotics on leptospirosis, we found a newly conducted meta-analysis involving 920 patients and 8 antibiotics. The analysis revealed that six antibiotics resulted in significantly shorter defervescence times compared to the control group. These antibiotics include cefotaxime, azithromycin, doxycycline, ceftriaxone, penicillin, and penicillin or ampicillin. However, antibiotics were not found to be effective in reducing mortality or hospital stays [31].

Lastly, concerning factors influencing leptospiral outcomes, we conducted our own meta-analysis of clinical predictors involving 1714 patients with leptospirosis. We found that patients with severe outcomes were more likely to experience dyspnea, oliguria, and hemorrhagic symptoms compared to non-severe patients [9].

## 5. Limitations

Despite the important findings from our retrospective cohort study on the impact of antibiotic and steroid therapy on leptospirosis outcomes in Transcarpathia, Ukraine, several limitations should be acknowledged. The study’s single-center design may constrain the generalizability of our findings to other regions or healthcare settings, as variability in diagnostic and treatment protocols across different centers could affect patient outcomes. Additionally, the retrospective nature of our research introduces inherent limitations, including the potential for incomplete or inaccurate medical records. Reliance on existing data also restricts our ability to control for all potential confounding variables.

With a sample size of 38 patients, although deemed sufficient through power analysis, the relatively small cohort may impact the robustness of our findings and limit the detection of smaller yet clinically relevant differences. While we attempted to control for several confounding variables, unmeasured factors may still influence outcomes, such as patient adherence to treatment, variations in supportive care, and socio-economic factors.

Future research should consider multicenter studies with larger sample sizes and prospective designs to validate and build upon our findings. Standardizing treatment protocols and thoroughly documenting potential confounders would further enhance the reliability and applicability of future results.

## 6. Conclusions

We observed that dexamethasone had a statistically significant effect on certain clinical variables, such as LYM, MID, GRA, platelet levels, AST, direct bilirubin, and urea levels. Similarly, cephalosporins showed significant effects on LYM, MID, platelet, AST, total bilirubin, direct bilirubin, and urea levels. Notably, direct bilirubin levels emerged as one of the strongest predictors of death in leptospirosis, exhibiting high sensitivity and specificity. These findings have implications for clinicians not only in the Transcarpathian region but also for practitioners worldwide.

## Figures and Tables

**Table 1:** Demographic and clinical characteristics of patients with leptospirosis in the Transcarpathian region of Ukraine.

	Abs.	%
Sex		
Male	27	71.1
Female	11	28.9
Serogroups		
Hebdomadis	10	26.3
Canicola	4	10.5
Clinical	3	7.9
Others	2	5.3
Pomona	3	7.9
Sejroe	5	13.2
Icterrohaemorrhagiae	3	7.9
Cynopteri	7	18.4
Australis	1	2.6
Outcome		
Survive	35	92.1
Non-survive	3	7.9
Antibiotic		
Cephalosporins	17	44.7
Benzylpenicillins	8	21.1
Others	13	34.2
Steroids		
None	3	7.9
Dexamethasone	29	76.3
Methylprednisolone	2	5.3
Others	4	10.5

**Table 2:** Clinical and laboratory findings at admission for patients with leptospirosis.

Variables	Median (Me)	Q₁–Q₃ (Interquartile Range)
Age (years)	50.00	44.25–63.75
Length of Stay (LoS) (days)	10.00	7.00–11.75
WBC (×10^9^/L)	10.73	6.87–14.81
LYM (×10^9^/L)	0.71	0.43–1.45
MID (×10^9^/L)	0.31	0.14–0.63
GRA (×10^9^/L)	8.18	6.25–13.38
ESR (mm/h)	46.50	30.00–75.25
RBC (×10^12^/L)	4.38	4.08–4.91
HGB (g/L)	128.00	113.50–140.75
MCV (fL)	89.50	87.00–92.00
PLT (×10^9^/L)	116.50	68.00–181.25
ALT (U/L)	92.15	55.00–128.85
AST (U/L)	76.15	44.52–123.35
Serum Creatinine (µmol/L)	138.90	112.67–421.27
Glucose (mmol/L)	7.28	6.55–9.04
GGT (U/L)	85.75	52.62–157.88
Total Bilirubin (µmol/L)	34.00	12.47–121.80
Direct Bilirubin (µmol/L)	19.35	6.22–91.58
Total Protein (g/L)	61.20	55.25–63.27
Urea (mmol/L)	10.82	8.26–23.08

**Table 3:** Effect of antibiotics on laboratory findings.

Variables	Group	On Admission (Median [Q1–Q3])	On Discharge (Median [Q1–Q3])	*p* Value
WBC (×10^9^/L)	Cephalosporins	8.39 (6.59–14.01)	9.25 (7.86–12.86)	0.747
	Benzylpenicillins	9.87 (7.49–12.73)	8.96 (6.77–11.04)	0.844
	Others	11.68 (7.12–18.00)	9.62 (7.31–12.08)	0.414
LYM (×10^9^/L)	Cephalosporins	0.62 (0.39–1.20)	2.50 (1.68–3.02)	0.002 *
	Benzylpenicillins	0.60 (0.43–0.86)	1.73 (1.25–1.91)	0.039 *
	Others	1.18 (0.53–2.14)	2.05 (1.58–2.81)	0.033 *
MID (×10^9^/L)	Cephalosporins	0.24 (0.14–0.39)	0.68 (0.43–0.81)	0.027 *
	Benzylpenicillins	0.34 (0.29–0.51)	0.84 (0.54–1.09)	0.028 *
	Others	0.36 (0.13–0.99)	0.71 (0.62–0.75)	0.497
GRA (×10^9^/L)	Cephalosporins	7.39 (6.15–12.16)	6.73 (5.08–8.71)	0.109
	Benzylpenicillins	8.46 (6.70–11.77)	6.03 (5.00–7.95)	0.250
	Others	8.54 (6.25–15.20)	6.94 (4.70–7.87)	0.094
ESR (mm/hr)	Cephalosporins	40.00 (27.00–63.00)	33.00 (20.00–60.00)	0.378
	Benzylpenicillins	63.50 (31.50–79.25)	51.50 (34.50–75.25)	0.641
	Others	59.00 (41.00–82.00)	49.00 (41.00–70.00)	0.127
RBC (×10^12^/L)	Cephalosporins	4.48 (4.24–5.01)	4.33 (3.89–4.93)	0.207
	Benzylpenicillins	4.01 (3.80–4.80)	3.67 (3.56–4.07)	0.039 *
	Others	4.38 (4.16–4.80)	4.37 (3.46–4.50)	0.008 *
HGB (g/L)	Cephalosporins	130.00 (123.00–140.00)	125.00 (116.00–144.00)	0.782
	Benzylpenicillins	121.50 (112.75–127.25)	115.50 (112.00–117.75)	0.028 *
	Others	127.00 (110.00–142.00)	127.00 (102.00–138.00)	0.340
MCV (fL)	Cephalosporins	90.00 (87.00–92.00)	92.00 (89.00–94.00)	0.006 *
	Benzylpenicillins	90.00 (86.75–93.00)	91.50 (88.50–95.25)	0.250
	Others	89.00 (87.00–95.00)	90.00 (88.00–97.00)	0.033 *
PLT (×10^9^/L)	Cephalosporins	136.00 (73.00–229.00)	346.00 (293.00–398.00)	0.003 *
	Benzylpenicillins	85.00 (49.75–150.50)	174.50 (132.25–338.50)	0.039 *
	Others	107.00 (68.00–139.00)	278.00 (228.00–339.00)	<0.001 *
ALT (U/L)	Cephalosporins	93.00 (41.60–169.30)	85.40 (64.40–106.80)	0.159
	Benzylpenicillins	85.65 (68.72–247.22)	68.50 (62.27–132.75)	0.250
	Others	85.30 (53.80–120.60)	55.90 (41.10–98.50)	0.839
AST (U/L)	Cephalosporins	81.30 (39.10–135.80)	49.30 (34.50–58.10)	0.015 *
	Benzylpenicillins	69.80 (48.12–156.90)	49.15 (27.60–93.25)	0.109
	Others	76.20 (44.10–103.00)	37.50 (27.60–53.90)	0.168
Serum Creatinine (µmol/L)	Cephalosporins	119.70 (107.10–243.80)	105.90 (102.90–126.40)	0.109
	Benzylpenicillins	264.80 (124.55–524.65)	117.75 (103.17–262.07)	0.461
	Others	144.10 (115.30–437.20)	109.90 (101.20–132.50)	<0.001 *
Glucose (mmol/L)	Cephalosporins	6.59 (6.45–7.77)	6.40 (6.00–6.76)	0.045*
	Benzylpenicillins	8.47 (6.75–9.48)	6.37 (5.88–7.86)	0.109
	Others	7.80 (6.77–9.10)	6.78 (6.53–8.91)	0.376
GGT (U/L)	Cephalosporins	120.00 (57.80–172.70)	79.90 (49.10–157.60)	0.064
	Benzylpenicillins	77.25 (50.67–123.05)	76.70 (45.30–110.97)	0.148
	Others	74.80 (47.60–125.60)	74.80 (61.70–110.00)	0.735
Total Bilirubin (µmol/L)	Cephalosporins	25.90 (10.80–56.60)	12.40 (11.10–29.20)	0.009 *
	Benzylpenicillins	108.66 (44.82–261.20)	43.35 (31.35–176.55)	0.612
	Others	20.50 (14.70–159.30)	20.10 (12.70–38.80)	0.080
Direct bilirubin (µmol/L)	Cephalosporins	14.40 (5.68–37.10)	7.80 (5.40–19.40)	0.015 *
	Benzylpenicillins	72.81 (31.20–170.40)	24.30 (17.12–99.60)	0.461
	Others	11.10 (6.90–99.20)	9.80 (6.90–20.50)	0.048 *
Total protein (g/L)	Cephalosporins	58.53 (54.70–63.30)	60.50 (58.60–62.30)	0.469
	Benzylpenicillins	59.75 (56.23–63.06)	59.53 (54.25–61.73)	0.641
	Others	62.30 (59.60–63.20)	61.80 (58.80–63.00)	0.244
Urea (mmol/L)	Cephalosporins	9.63 (8.22–20.39)	7.35 (6.52–8.18)	0.013 *
	Benzylpenicillins	20.52 (8.12–30.87)	9.45 (7.56–18.40)	0.945
	Others	17.45 (8.72–23.02)	7.92 (6.65–8.63)	0.010 *

Asterisk (*) indicates statistically significant changes between admission and discharge.

## Data Availability

The raw data supporting the conclusions of this article will be made available by the authors upon request.
